# Clostridial Butyrate Biosynthesis Enzymes Are Significantly Depleted in the Gut Microbiota of Nonobese Diabetic Mice

**DOI:** 10.1128/mSphere.00492-18

**Published:** 2018-10-24

**Authors:** Alessandro Tanca, Antonio Palomba, Cristina Fraumene, Valeria Manghina, Michael Silverman, Sergio Uzzau

**Affiliations:** a Porto Conte Ricerche, Tramariglio, Alghero, Italy; b Dipartimento di Scienze Biomediche, Università di Sassari, Sassari, Italy; c Division of Immunology, Department of Microbiology and Immunobiology, Harvard Medical School, Boston, Massachusetts, USA; d Division of Infectious Diseases, Department of Medicine, Boston Children’s Hospital, Boston, Massachusetts, USA; Arizona State University

**Keywords:** butyrate, diabetes, metaproteomics, microbiome, short-chain fatty acids

## Abstract

Increasing evidence suggests that the intestinal microbiota is involved in the pathogenesis of type 1 diabetes (T1D). Here we sought to determine which gut microbial taxa and functions vary between nonobese diabetic (NOD) mice and genetically modified NOD mice protected from T1D (Eα16/NOD) at 10 weeks of age in the time window between insulitis development and T1D onset. The gut microbiota of NOD mice were investigated by analyzing stool samples with a metaproteogenomic approach, comprising both 16S rRNA gene sequencing and microbial proteome profiling through high-resolution mass spectrometry. A depletion of *Firmicutes* (particularly, several members of *Lachnospiraceae*) in the NOD gut microbiota was observed compared to the level in the Eα16/NOD mice microbiota. Moreover, the analysis of proteins actively produced by the gut microbiota revealed different profiles between NOD and Eα16/NOD mice, with the production of butyrate biosynthesis enzymes being significantly reduced in diabetic mice. Our results support a model for gut microbiota influence on T1D development involving bacterium-produced metabolites as butyrate.

**IMPORTANCE** Alterations of the gut microbiota early in age have been hypothesized to impact T1D autoimmune pathogenesis. In the NOD mouse model, protection from T1D has been found to operate via modulation of the composition of the intestinal microbiota during a critical early window of ontogeny, although little is known about microbiota functions related to T1D development. Here, we show which gut microbial functions are specifically associated with protection from T1D in the time window between insulitis development and T1D onset. In particular, we describe that production of butyrate biosynthesis enzymes is significantly reduced in NOD mice, supporting the hypothesis that modulating the gut microbiota butyrate production may influence T1D development.

## Abstract (precis)

Alterations of the gut microbiota early in age have been hypothesized to impact T1D autoimmune pathogenesis. In the NOD mouse model, protection from T1D has been found to operate via modulation of the composition of the intestinal microbiota during a critical early window of ontogeny, although little is known about microbiota functions related to T1D development. Here, we show which gut microbial functions are specifically associated with protection from T1D in the time window between insulitis development and T1D onset. In particular, we describe that production of butyrate biosynthesis enzymes is significantly reduced in NOD mice, supporting the hypothesis that modulating the gut microbiota butyrate production may influence T1D development.

## OBSERVATION

Type 1 diabetes (T1D) is an autoimmune disease characterized by the specific destruction of pancreatic insulin-producing β-cells (1). Although there is evidence of a strong genetic basis for T1D (2), a remarkable increase in T1D incidence has been measured in the last decades, suggesting a significant contribution from the environment (3). Among possible environmental factors influencing T1D development, many may be significantly related to the gut microbiota, including hygiene, antibiotic use, and diet (4). Gut microbial colonization starts at birth, playing a key role in priming the immune system (5), as well as in complementing the host's metabolism (6). Alterations of the gut microbiota during host development have been hypothesized to have an impact on T1D autoimmune pathogenesis (7), although a full comprehension of the host-microbiota interactions related to T1D is far from being achieved.

The most widely studied animal model of autoimmune diabetes is the nonobese diabetic (NOD) mouse. Diabetes in NOD mice is a T cell-dependent disorder, with the major histocompatibility complex (MHC) locus being the dominant genetic determinant, as in human T1D (8). NOD mice do not express the MHC class II E complex due to deletion of the Eα promoter (9), and genetically modified NOD mice expressing the Eα molecule are completely protected from T1D (10). We recently demonstrated that this protection operates via modulation of the composition of the intestinal microbiota during a critical early window of ontogeny (11). In particular, we observed that the microbiota of Eα16/NOD mice (protected from T1D) starts to differentiate from that of NOD mice, both in terms of alpha- and beta-diversity, before initiation of insulitis.

Here, we carried out a deep metaproteogenomic characterization of fecal microbiota collected from 10-week-old Eα16/NOD and NOD mice (6 Eα16/NOD mice versus 6 NOD mice) in order to identify which microbial functions specifically discriminate the two microbiotas before T1D onset and therefore possibly correlate to T1D protection/development. Materials and methods are detailed in Text S1 in the supplemental material.

10.1128/mSphere.00492-18.4 TEXT S1 Supplemental methods. Download Text S1, DOCX file, 0.02 MB. Copyright © 2018 Tanca et al. 2018 Tanca et al. This content is distributed under the terms of the Creative Commons Attribution 4.0 International license.

Metaproteomic (MP) analyses led to the detection of 153,044 microbial peptide-spectrum matches, mapped to 12,790 microbial sequences, and assigned to 1,205 functions and 448 taxa/243 genera. In parallel, 2,139,608 reads were obtained upon 16S rRNA gene sequencing (16S), mapped to 14,022 operational taxonomic units (OTUs), and assigned to 639 taxa/297 genera.

**FIG 1:**
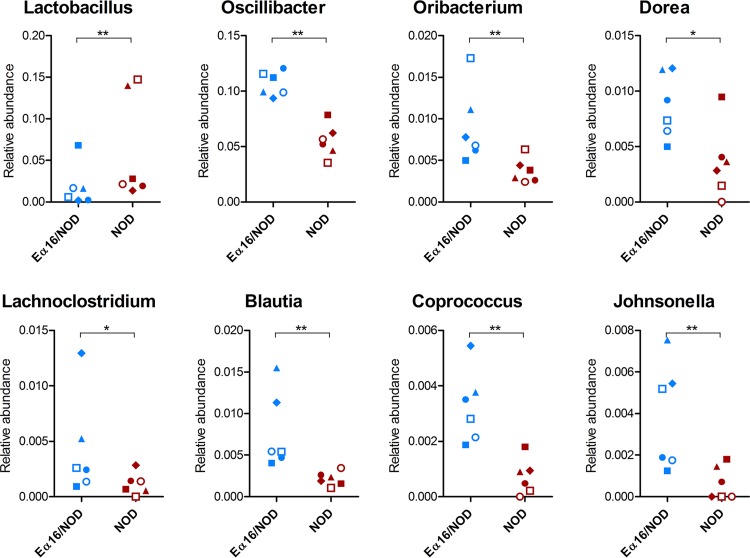
Microbial genera showing significantly differential abundances between Eα16/NOD and NOD mouse fecal metaproteomes. Genera detected in all samples of at least one group are shown. Each dot represents a different mouse, with Eα16/NOD and NOD littermates reared in the same cage having the same shape. A paired sample test based on an inverted beta binomial model was used, with each pair of samples comprising two littermates with different genotypes. *, *q* value < 0.05; **, *q* value < 0.01.

First, we sought for taxonomic differences in the gut metaproteome profiles between Eα16/NOD and NOD mice. The application of a paired sample test (taking into account the cage effect, with each cage housing two littermates with different genotypes) enabled the identification of 19 differential taxa (Data Set S1). A global depletion of *Firmicutes* proteins was found in the NOD mice microbiota compared to the level in Eα16/NOD mice, mainly attributable to the order *Clostridiales*. At lower levels, 7 clostridial genera, namely, *Blautia*, *Coprococcus*, *Dorea*, *Johnsonella*, *Lachnoclostridium*, *Oribacterium* from *Lachnospiraceae*, and *Oscillibacter* from *Oscillospiraceae*, were detected with significantly lower abundances in the NOD mouse fecal microbiota, as displayed in Fig. 1. On the other hand, *Lactobacillus* proteins were found to be significantly higher in abundance in the NOD metaproteome than in the Eα16/NOD counterpart. MP results were globally consistent with 16S data (Data Set S2), with the large majority of differential OTUs and genera that were more abundant in Eα16/NOD mice being assigned to the *Clostridiales* (Fig. S1 and S2). Interestingly, all OTUs higher in abundance in NOD mice were classified as belonging to the order *Bacteroidales*. Our taxonomic results are consistent with those of previous investigations carried out with human subjects at the onset of T1D. Specifically, several 16S rRNA studies of the gut microbiota from children matched for age, sex, early feeding history, and human leukocyte antigen risk genotype and differing in T1D-associated autoantibodies demonstrated a striking depletion of butyrate-producing clostridial species and an increase in *Bacteroidetes* spp. in children with β-cell autoimmunity (12–15).

10.1128/mSphere.00492-18.1 FIG S1 Bar graph illustrating the distribution of differential OTUs based on their taxonomic annotation (red, taxa higher in NOD mice microbiotas; blue, taxa higher in Eα16/NOD mice microbiotas). A paired sample (inverted beta binomial) test was applied to find the differential OTUs. Download FIG S1, TIF file, 1.4 MB. Copyright © 2018 Tanca et al. 2018 Tanca et al. This content is distributed under the terms of the Creative Commons Attribution 4.0 International license.

10.1128/mSphere.00492-18.2 FIG S2 Clostridial genera showing significantly differential abundances between Eα16/NOD and NOD mouse fecal microbiotas upon 16S rRNA gene analysis. Each dot represents a different mouse, with Eα16/NOD and NOD littermates reared in the same cage having the same shape. A paired sample test based on an inverted beta binomial model was used, with each pair of samples comprising two littermates with different genotype. *, *q* value < 0.05; **, *q* value < 0.01. Download FIG S2, TIF file, 0.6 MB. Copyright © 2018 Tanca et al. 2018 Tanca et al. This content is distributed under the terms of the Creative Commons Attribution 4.0 International license.

10.1128/mSphere.00492-18.3 DATA SET S1 Data set containing identification, annotation, and differential analysis information from metaproteomic data. Download Data Set S1, XLSX file, 2.1 MB. Copyright © 2018 Tanca et al. 2018 Tanca et al. This content is distributed under the terms of the Creative Commons Attribution 4.0 International license.

10.1128/mSphere.00492-18.4 DATA SET S2 Data set containing identification, annotation, and differential analysis information from 16S rRNA gene sequencing data. Download Data Set S2, XLSX file, 1.4 MB. Copyright © 2018 Tanca et al. 2018 Tanca et al. This content is distributed under the terms of the Creative Commons Attribution 4.0 International license.

We then focused on the protein functions expressed by the members of the microbiota, as enabled by the MP approach. As a result, we found 59 functions with significantly differential abundances between the microbiotas of NOD and Eα16/NOD mice (Data Set S1). When associating phylum and function information, we found 105 phylum-specific functions showing differential abundances between the two genotypes (Data Set S1); interestingly, all 87 of those organisms more abundant in the microbiota of Eα16/NOD mice were associated with *Firmicutes*, while most of those more abundant in the microbiota of NOD mice belonged to *Bacteroidetes*. Protein functions that were more abundant in the NOD mouse microbiota and assigned to *Bacteroidetes* were implicated primarily in polysaccharide transport and degradation. Intriguingly, we observed the increase of a few *Firmicutes* proteins in the NOD mouse microbiota (a trend opposite to that of all other proteins from the same phylum), namely, a small acid-soluble spore protein, enolase, and a cell surface glycoprotein (S-layer protein), all possibly related to bacterial survival and host immune evasion (16), and phosphoketolase. On the other hand, proteins specifically enriched in the microbiotas of T1D-protected mice (mostly from the *Clostridia*) were related to many different activities, including enzymes involved in acetogenesis (carbon monoxide dehydrogenase), butyrogenesis (see below), pyruvate metabolism (including pyruvate synthase), polysaccharide metabolism (e.g., glycogen synthase), and amino acid biosynthesis (such as cysteine and tryptophan synthases), as well as proteins associated with carbohydrate transport, response to stress, and chemotaxis.

**FIG 2:**
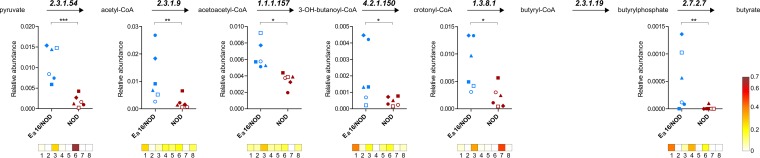
Expression of butyrate biosynthesis enzymes in the fecal microbiotas of Eα16/NOD and NOD mice according to metaproteomic results. (Top) Schematic of butyrate biosynthetic pathway (from pyruvate to butyrate), with Enzyme Commission numbers corresponding to each enzyme. EC 2.3.1.54, formate acetyltransferase; EC 2.3.1.9, acetyl-CoA acetyltransferase; EC 1.1.1.157, 3-hydroxybutyryl-CoA dehydrogenase; EC 4.2.1.150, short-chain-enoyl-CoA hydratase; EC 1.3.8.1, butyryl-CoA dehydrogenase; EC 2.3.1.19, phosphate butyryltransferase; EC 2.7.2.7, butyrate kinase. (Middle) Scatterplots illustrating the relative abundance of each butyrogenic enzyme in the fecal microbiotas of Eα16/NOD and NOD mice; each dot represents a different mouse, with Eα16/NOD and NOD littermates reared in the same cage having the same shape. A paired sample test based on an inverted beta binomial model was used, with each pair of samples comprising two littermates with different genotypes. *, *q* value < 0.05; **, *q* value < 0.01; ***, *q* value < 0.001. (Bottom) Heatmaps showing the relative distribution of the taxonomic assignments (according to the lowest-common-ancestor principle) associated with each enzyme. Taxa associated with at least one enzyme and found in at least 4 mice are reported. Boxes: 1, *Firmicutes*; 2, *Firmicutes*, *Clostridia*; 3, *Firmicutes*, *Clostridia*, *Clostridiales*; 4, *Firmicutes*, *Clostridia*, *Clostridiales*, *Clostridiaceae*, *Clostridium*; 5, *Firmicutes*, *Clostridia*, *Clostridiales*, *Eubacteriaceae*, *Eubacterium*; 6, *Firmicutes*, *Clostridia*, *Clostridiales, Lachnospiraceae*; 7, *Firmicutes*, *Clostridia*, *Clostridiales*, *Lachnospiraceae*, *Roseburia*; 8, *Firmicutes*, *Clostridia*, *Clostridiales*, *Oscillospiraceae*, *Oscillibacter*.

Remarkably, we found that all key enzymes involved in butyrate biosynthesis and expressed by members of the *Clostridia* were significantly depleted in the gut microbiotas of NOD mice, compared to those of T1D-protected Eα16/NOD mice. Figure 2 shows the expression profiles of six butyrogenic enzymes (mapping almost entirely to the butyrate biosynthesis pathway, from pyruvate to butyrate via butyrate kinase), along with the taxonomic assignments of the related peptides. As a result, the increase of butyrogenic activity in the microbiotas of T1D-protected mice was revealed to be due mainly to members of the *Clostridiales*, including *Clostridium*, *Eubacterium*, *Roseburia*, and *Oscillibacter*. Throughout the metabolic pathway, some catalytic functions were found to be family or even genus specific, while others were shared by several members of the microbiota or could not be mapped at the lowest taxonomic levels, being highly conserved sequences. Butyrate represents the main source of energy for enterocytes and is able to enhance mucus production in the gut, thus having a profound impact on the integrity of the gut barrier (3). Impaired butyrate production provides a possible explanation for the loss of tight barrier function which has been related to T1D pathogenesis according to the so-called "leaky gut" hypothesis (17). Additionally, butyrate is known to contribute significantly to the induction of colonic regulatory T cells (18, 19) and has been demonstrated to exert anti-inflammatory effects via several mechanisms, including histone deacetylase (20) and NF-κB (21) inhibition.

Indirect evidence that microbial production of butyrate also exerts an important role on preventing T1D was previously reported with different experimental approaches. The proportion of butyryl coenzyme A (butyryl-CoA) dehydrogenase genes was shown to be lower in the metagenomes of T1D cases than in those of healthy controls (12). Another study reanalyzed 16S rRNA data from 6-month-old children of a prospective cohort by applying microbial cooccurrence networks, suggesting a protective role of butyrate in T1D pathogenesis (22). Moreover, the direct administration of short-chain fatty acids (SCFAs) or SCFA-yielding diets to rodent models of T1D was proven to provide a high degree of protection from T1D-related autoimmune responses (23, 24). Specifically, butyrate was demonstrated to promote the abundance and the function of T regulatory cells.

In conclusion, we report here for the first time that butyrate biosynthesis enzymes are depleted in a T1D model. Hence, our data further support the hypothesis of involvement of SCFA production in T1D pathogenesis and specifically underline a possible key role of butyrate in the complex series of events leading to autoimmune insulitis and preceding T1D onset.

### Data availability.

16S rRNA gene sequencing data have been deposited into the European Nucleotide Archive with the study accession number PRJEB25325.

The mass spectrometry proteomics data (including the above-mentioned sequence database) have been deposited to the ProteomeXchange Consortium via the PRIDE (25) partner repository with the data set identifier PXD003616.

## References

[1] Atkinson MA, Eisenbarth GS, Michels AW 2014 Type 1 diabetes. Lancet 383:69–82. doi:10.1016/S0140-6736(13)60591-7. 23890997 PMC4380133

[2] Eringsmark Regnell S, Lernmark A 2013 The environment and the origins of islet autoimmunity and type 1 diabetes. Diabet Med 30:155–160. doi:10.1111/dme.12099. 23252770 PMC3552102

[3] Endesfelder D, Engel M, Zu Castell W 2016 Gut immunity and type 1 diabetes: a melange of microbes, diet, and host interactions? Curr Diab Rep 16:60. doi:10.1007/s11892-016-0753-3. 27155610

[4] Knip M, Simell O 2012 Environmental triggers of type 1 diabetes. Cold Spring Harb Perspect Med 2:a007690. doi:10.1101/cshperspect.a007690. 22762021 PMC3385937

[5] Belkaid Y, Hand TW 2014 Role of the microbiota in immunity and inflammation. Cell 157:121–141. doi:10.1016/j.cell.2014.03.011. 24679531 PMC4056765

[6] Krishnan S, Alden N, Lee K 2015 Pathways and functions of gut microbiota metabolism impacting host physiology. Curr Opin Biotechnol 36:137–145. doi:10.1016/j.copbio.2015.08.015. 26340103 PMC4688195

[7] Needell JC, Zipris D 2016 The role of the intestinal microbiome in type 1 diabetes pathogenesis. Curr Diab Rep 16:89. doi:10.1007/s11892-016-0781-z. 27523648

[8] Pearson JA, Wong FS, Wen L 2016 The importance of the non obese diabetic (NOD) mouse model in autoimmune diabetes. J Autoimmun 66:76–88. doi:10.1016/j.jaut.2015.08.019. 26403950 PMC4765310

[9] Mathis DJ, Benoist C, Williams VE II, Kanter M, McDevitt HO 1983 Several mechanisms can account for defective E alpha gene expression in different mouse haplotypes. Proc Natl Acad Sci U S A 80:273–277. doi:10.1073/pnas.80.1.273. 6296871 PMC393355

[10] Bohme J, Schuhbaur B, Kanagawa O, Benoist C, Mathis D 1990 MHC-linked protection from diabetes dissociated from clonal deletion of T cells. Science 249:293–295. doi:10.1126/science.2115690. 2115690

[11] Silverman M, Kua L, Tanca A, Pala M, Palomba A, Tanes C, Bittinger K, Uzzau S, Benoist C, Mathis D 2017 Protective major histocompatibility complex allele prevents type 1 diabetes by shaping the intestinal microbiota early in ontogeny. Proc Natl Acad Sci U S A 114:9671–9676. doi:10.1073/pnas.1712280114. 28831005 PMC5594701

[12] Brown CT, Davis-Richardson AG, Giongo A, Gano KA, Crabb DB, Mukherjee N, Casella G, Drew JC, Ilonen J, Knip M, Hyoty H, Veijola R, Simell T, Simell O, Neu J, Wasserfall CH, Schatz D, Atkinson MA, Triplett EW 2011 Gut microbiome metagenomics analysis suggests a functional model for the development of autoimmunity for type 1 diabetes. PLoS One 6:e25792. doi:10.1371/journal.pone.0025792. 22043294 PMC3197175

[13] Giongo A, Gano KA, Crabb DB, Mukherjee N, Novelo LL, Casella G, Drew JC, Ilonen J, Knip M, Hyoty H, Veijola R, Simell T, Simell O, Neu J, Wasserfall CH, Schatz D, Atkinson MA, Triplett EW 2011 Toward defining the autoimmune microbiome for type 1 diabetes. ISME J 5:82–91. doi:10.1038/ismej.2010.92. 20613793 PMC3105672

[14] de Goffau MC, Fuentes S, van den Bogert B, Honkanen H, de Vos WM, Welling GW, Hyöty H, Harmsen HJM 2014 Aberrant gut microbiota composition at the onset of type 1 diabetes in young children. Diabetologia 57:1569–1577. doi:10.1007/s00125-014-3274-0. 24930037

[15] de Goffau MC, Luopajarvi K, Knip M, Ilonen J, Ruohtula T, Harkonen T, Orivuori L, Hakala S, Welling GW, Harmsen HJ, Vaarala O 2013 Fecal microbiota composition differs between children with beta-cell autoimmunity and those without. Diabetes 62:1238–1244. doi:10.2337/db12-0526. 23274889 PMC3609581

[16] Henderson B, Martin A 2011 Bacterial virulence in the moonlight: multitasking bacterial moonlighting proteins are virulence determinants in infectious disease. Infect Immun 79:3476–3491. doi:10.1128/IAI.00179-11. 21646455 PMC3165470

[17] Vaarala O 2008 Leaking gut in type 1 diabetes. Curr Opin Gastroenterol 24:701–706. doi:10.1097/MOG.0b013e32830e6d98. 19122519

[18] Furusawa Y, Obata Y, Fukuda S, Endo TA, Nakato G, Takahashi D, Nakanishi Y, Uetake C, Kato K, Kato T, Takahashi M, Fukuda NN, Murakami S, Miyauchi E, Hino S, Atarashi K, Onawa S, Fujimura Y, Lockett T, Clarke JM, Topping DL, Tomita M, Hori S, Ohara O, Morita T, Koseki H, Kikuchi J, Honda K, Hase K, Ohno H 2013 Commensal microbe-derived butyrate induces the differentiation of colonic regulatory T cells. Nature 504:446–450. doi:10.1038/nature12721. 24226770

[19] Smith PM, Howitt MR, Panikov N, Michaud M, Gallini CA, Bohlooly YM, Glickman JN, Garrett WS 2013 The microbial metabolites, short-chain fatty acids, regulate colonic Treg cell homeostasis. Science 341:569–573. doi:10.1126/science.1241165. 23828891 PMC3807819

[20] Chang PV, Hao L, Offermanns S, Medzhitov R 2014 The microbial metabolite butyrate regulates intestinal macrophage function via histone deacetylase inhibition. Proc Natl Acad Sci U S A 111:2247–2252. doi:10.1073/pnas.1322269111. 24390544 PMC3926023

[21] Inan MS, Rasoulpour RJ, Yin L, Hubbard AK, Rosenberg DW, Giardina C 2000 The luminal short-chain fatty acid butyrate modulates NF-kappaB activity in a human colonic epithelial cell line. Gastroenterology 118:724–734. doi:10.1016/S0016-5085(00)70142-9. 10734024

[22] Endesfelder D, Engel M, Davis-Richardson AG, Ardissone AN, Achenbach P, Hummel S, Winkler C, Atkinson M, Schatz D, Triplett E, Ziegler AG, zu Castell W 2016 Towards a functional hypothesis relating anti-islet cell autoimmunity to the dietary impact on microbial communities and butyrate production. Microbiome 4:17. doi:10.1186/s40168-016-0163-4. 27114075 PMC4845316

[23] Needell JC, Ir D, Robertson CE, Kroehl ME, Frank DN, Zipris D 2017 Maternal treatment with short-chain fatty acids modulates the intestinal microbiota and immunity and ameliorates type 1 diabetes in the offspring. PLoS One 12:e0183786. doi:10.1371/journal.pone.0183786. 28886045 PMC5590848

[24] Marino E, Richards JL, McLeod KH, Stanley D, Yap YA, Knight J, McKenzie C, Kranich J, Oliveira AC, Rossello FJ, Krishnamurthy B, Nefzger CM, Macia L, Thorburn A, Baxter AG, Morahan G, Wong LH, Polo JM, Moore RJ, Lockett TJ, Clarke JM, Topping DL, Harrison LC, Mackay CR 2017 Gut microbial metabolites limit the frequency of autoimmune T cells and protect against type 1 diabetes. Nat Immunol 18:552–562. doi:10.1038/ni.3713. 28346408

[25] Vizcaino JA, Csordas A, Del-Toro N, Dianes JA, Griss J, Lavidas I, Mayer G, Perez-Riverol Y, Reisinger F, Ternent T, Xu QW, Wang R, Hermjakob H 2016 2016 update of the PRIDE database and its related tools. Nucleic Acids Res 44:D447–D456. doi:10.1093/nar/gkv1145. 26527722 PMC4702828

