# Supplementary material for: Clostridial Butyrate Biosynthesis Enzymes Are Significantly Depleted in the Gut Microbiota of Nonobese Diabetic Mice
Source: mSphere. 2018 Oct 24;3(5):e00492-18. doi: 10.1128/mSphere.00492-18 (PMC6200989; doi:10.1128/mSphere.00492-18)
Supplement: TEXT S1 [file sph006182677s1.docx]

**Text S1**

**Mice**

Eα16/non-obese diabetic (NOD) mice were maintained at the Jackson Laboratory, and imported into the specific pathogen-free facility at the New Research Building at Harvard Medical School for breeding. Mice were provided a standard chow diet *ad libitum* (PicoLab Mouse Diet 20, #5058, LabDiet, St. Louis, MO, USA). Mice were maintained free of segmented filamentous bacteria, as confirmed by PCR testing of mouse feces (1). Litters were weaned at 18-21 days of age. NOD and Eα16/NOD littermates remained cohoused (6 cages, each containing 1 NOD and 1 Eα16/NOD littermates). Fresh fecal pellets (approximately 100 mg for each mouse) were collected at 10 weeks of age into sterile tubes under a laminar flow hood, immediately placed on dry ice and then stored at -80°C until processing. Fecal samples (6 NOD and 6 Eα16/NOD) were sent in dry ice to Porto Conte Ricerche laboratories, where they were thawed at 4°C; two portions were collected from each sample for DNA and protein extraction, respectively.

This study was carried out in accordance with the recommendations of the Institutional Animal Care and Use Committee of Harvard Medical School, and the experimental protocol was approved by the same Committee. All experiments were performed in accordance with relevant regulatory standards.

**DNA extraction and 16S rRNA gene sequencing**

DNA extraction was carried out using the QIAamp DNA Stool Mini Kit (Qiagen, Hilden, Germany), according to the manufacturer's protocol. Amplification of the entire 16S-rRNA genes was performed using the universal primers 27F-1492R (AGAGTTTGATYMTGGCTCAG and TACGGYTACCTTGTTACGACTT, respectively) and the recombinant Taq DNA Polymerase from Invitrogen (Thermo Scientific, San Jose, CA, USA). PCR cycling conditions were as follows: 2 minutes at 94°C; 28 cycles of 30 seconds at 94°C, 30 seconds at 55°C, 2 minutes at 68°C; finally, 7 minutes at 72°C. PCR products were confirmed on 2% agarose gel (Sigma Aldrich, St. Louis, MO, USA). The 16S rRNA gene amplification reaction was performed in duplicate, then the two amplification products were pooled together, cleaned up using AMPure XP (Beckman Coulter, Brea, CA) magnetic beads and quantified with the Qubit HS assay using the Qubit fluorimeter 2.0 (Life Technologies, Grand Island, NY, USA).

Libraries were constructed according to the Nextera XT kit (Illumina, San Diego, CA, USA). The average insert size was around 500 bps. Sequence-ready libraries were normalized to ensure equal library representation in the pooled samples. DNA sequencing was performed with the Illumina HiScanSQ sequencer, using the paired-end method and 93 cycles of sequencing.

**16S rRNA gene sequencing data bioinformatics**

The Illumina demultiplexed paired-reads were trimmed for the first 20 bps using FASTX and the sequences with Nextera adapter contamination were identified using the UniVec database (ftp://ftp.ncbi.nlm.nih.gov/pub/UniVec) and removed. Paired reads were merged using the script join_paired_ends.py inside the QIIME package, v.1.9.0 (2) with a minimum overlap of 8 base pairs. Operational taxonomic unit (OTU) generation was done using a QIIME pipeline based on USEARCH's OTU clustering recommendations (http://www.drive5.com/usearch/manual/otu_clustering.html) using the closed-reference OTU picking to allow clustering of shotgun 16S sequences. Reads were clustered at 97% identity using UCLUST to produce OTUs (3). Taxonomy assignment of resulting OTUs was performed using the Greengenes 13_8 database (4). With taxonomic lineages in hand, OTU tables were computed using QIIME (2, 5).

**Protein extraction and proteomic analysis**

Each sample was resuspended by vortexing in sodium dodecyl sulfate-based extraction buffer and then heated and subjected to a combination of bead-beating and freeze-boiling steps as detailed elsewhere (6). Protein extracts were subjected to on-filter reduction, alkylation, and trypsin digestion according to the filter-aided sample preparation (FASP) protocol (7), with slight modifications detailed elsewhere (8).

Liquid chromatography (LC)-tandem mass spectrometry (MS/MS) analysis was carried out using an LTQ-Orbitrap Velos mass spectrometer (Thermo Scientific) interfaced with an UltiMate 3000 RSLCnano LC system (Thermo Scientific). The single-run one-dimensional LC peptide separation was performed as previously described (6), loading 4 μg of peptide mixture per each sample and applying a 485 min separation gradient. The mass spectrometer was set up in a data dependent MS/MS mode, with Higher Energy Collision Dissociation as the fragmentation method, as detailed elsewhere (8). Samples were analyzed in randomized order.

**Metaproteomics bioinformatics**

Peptide identification was performed using the Proteome Discoverer informatic platform (version 2.0; Thermo Scientific), with Sequest-HT as search engine and Percolator for peptide validation (false discovery rate < 1%). Search parameters were set as follows: precursor mass threshold 350-5000 Da; minimum peak count 6; signal-to-noise threshold 2; enzyme trypsin; maximum missed cleavage sites 2; peptide length range 5-50 amino acids; precursor mass tolerance 10 ppm; fragment mass tolerance 0.02 Da; dynamic modification methionine oxidation; static modification cysteine carbamidomethylation. A collection of metagenomic sequences previously obtained in house from other NOD mouse fecal samples and processed according to previous reports (9) was merged with a previously published (10) and publicly available (ftp://penguin.genomics.cn/pub/10.5524/100001_101000/100114/Genecatalog/184sample_2.6M.GeneSet.pep.gz) mouse metagenomic dataset, and employed as sequence database for identification of microbial peptides (9,825,357 sequences in total). The mass spectra were also searched against a second database containing the *Mus musculus* proteome from UniProtKB (release 2017_08) in order to identify host proteins. In this latter case, protein grouping according to the strict parsimony principle was allowed. In total, 339 mouse proteins were identified, but none of them was found differential between NOD and Eα16/NOD mice in this study.

Taxonomic and functional annotation was performed using multiple strategies. MEGAN v.6.8.18 was used as first annotation option (11). Protein sequences were preliminary subjected to a DIAMOND (v.0.8.22) search against the NCBI-nr database (2016/09 update), using the blastp command with default parameters (12); then, DIAMOND outputs were loaded on MEGAN and both lowest common ancestor (LCA) classification and functional annotation (InterPro module) were performed using default parameters. Furthermore, the Unipept web application (v.3.2; https://unipept.ugent.be) was used to carry out an LCA classification of the identified peptide sequences (13). Finally, an additional functional annotation was accomplished by aligning the identified protein sequences against a database containing all bacterial sequences from UniProtKB/Swiss-Prot (release 2016_09) using DIAMOND (blastp module, e-value threshold 10^-5^); UniProtKB/Swiss-Prot accession numbers were subsequently exploited to retrieve protein name and Kyoto Encyclopedia of Genes and Genomes (KEGG) orthologous group information from the UniProt website via the 'retrieve' tool (14). Taxonomic information from different sources were combined, giving priority to MEGAN results; functional information from different sources were inspected, merged and made uniform manually. Peptides identified using the microbial database but assigned to non-microbial taxa were filtered out. In case of assignment of the same peptide to multiple metagenomic database sequences, only the first assignment was retained in order to avoid redundancies. Metaproteomic spectral count data obtained for each sample were then aggregated based on the various levels of annotation considered in the study (namely, phylum, class, order, family and genus as taxonomic annotation levels, and function, phylum-specific function, family-specific function and genus-specific function as functional annotation levels). The butyrate biosynthetic pathway was reconstructed based on the corresponding KEGG pathway map (15), available at http://www.genome.jp/kegg/pathway.html.

**Statistical analysis and graph generation**

Differential analysis of 16S rRNA gene sequencing data (read counts) and metaproteomic data (spectral counts) was performed using a paired sample test for count data based on an inverted beta binomial (ibb) model (16), according to previous reports (17). The paired sample test was chosen to take into account the well-known cage effect, with each pair of samples comprising two littermates with different genotype. Only features with >50% valid values in at least one group were included as input for the differential analysis. The p-value list provided by the ibb test was subsequently subjected to correction for multiple testing according to the Benjamini-Hochberg approach (18). A false discovery rate < 0.05 was considered as the threshold for statistical significance of differential results, and significant features detected in less than 80% of samples of the group with the highest abundance were filtered out.

Scatter plots were created with GraphPad Prism (v.5.03) starting from relative abundance data. Heatmaps were generated using the web application Morpheus (https://software.broadinstitute.org/morpheus).

**Supplemental references**

1. Kriegel MA, Sefik E, Hill JA, Wu HJ, Benoist C, Mathis D. 2011. Naturally transmitted segmented filamentous bacteria segregate with diabetes protection in nonobese diabetic mice. Proc Natl Acad Sci U S A 108:11548-11553.

2. Caporaso JG, Kuczynski J, Stombaugh J, Bittinger K, Bushman FD, Costello EK, Fierer N, Pena AG, Goodrich JK, Gordon JI, Huttley GA, Kelley ST, Knights D, Koenig JE, Ley RE, Lozupone CA, McDonald D, Muegge BD, Pirrung M, Reeder J, Sevinsky JR, Turnbaugh PJ, Walters WA, Widmann J, Yatsunenko T, Zaneveld J, Knight R. 2010. QIIME allows analysis of high-throughput community sequencing data. Nat Methods 7:335-336.

3. Edgar RC. 2010. Search and clustering orders of magnitude faster than BLAST. Bioinformatics 26:2460-2461.

4. DeSantis TZ, Hugenholtz P, Larsen N, Rojas M, Brodie EL, Keller K, Huber T, Dalevi D, Hu P, Andersen GL. 2006. Greengenes, a chimera-checked 16S rRNA gene database and workbench compatible with ARB. Appl Environ Microbiol 72:5069-5072.

5. Kuczynski J, Costello EK, Nemergut DR, Zaneveld J, Lauber CL, Knights D, Koren O, Fierer N, Kelley ST, Ley RE, Gordon JI, Knight R. 2010. Direct sequencing of the human microbiome readily reveals community differences. Genome Biol 11:210.

6. Tanca A, Palomba A, Pisanu S, Deligios M, Fraumene C, Manghina V, Pagnozzi D, Addis MF, Uzzau S. 2014. A straightforward and efficient analytical pipeline for metaproteome characterization. Microbiome 2:49.

7. Wisniewski JR, Zougman A, Nagaraj N, Mann M. 2009. Universal sample preparation method for proteome analysis. Nat Methods 6:359-362.

8. Tanca A, Biosa G, Pagnozzi D, Addis MF, Uzzau S. 2013. Comparison of detergent-based sample preparation workflows for LTQ-Orbitrap analysis of the *Escherichia coli* proteome. Proteomics 13:2597-2607.

9. Tanca A, Palomba A, Fraumene C, Pagnozzi D, Manghina V, Deligios M, Muth T, Rapp E, Martens L, Addis MF, Uzzau S. 2016. The impact of sequence database choice on metaproteomic results in gut microbiota studies. Microbiome 4:51.

10. Xiao L, Feng Q, Liang S, Sonne SB, Xia Z, Qiu X, Li X, Long H, Zhang J, Zhang D, Liu C, Fang Z, Chou J, Glanville J, Hao Q, Kotowska D, Colding C, Licht TR, Wu D, Yu J, Sung JJ, Liang Q, Li J, Jia H, Lan Z, Tremaroli V, Dworzynski P, Nielsen HB, Backhed F, Dore J, Le Chatelier E, Ehrlich SD, Lin JC, Arumugam M, Wang J, Madsen L, Kristiansen K. 2015. A catalog of the mouse gut metagenome. Nat Biotechnol 33:1103-1108.

11. Huson DH, Beier S, Flade I, Gorska A, El-Hadidi M, Mitra S, Ruscheweyh HJ, Tappu R. 2016. MEGAN Community Edition - Interactive Exploration and Analysis of Large-Scale Microbiome Sequencing Data. PLoS Comput Biol 12:e1004957.

12. Buchfink B, Xie C, Huson DH. 2015. Fast and sensitive protein alignment using DIAMOND. Nat Methods 12:59-60.

13. Mesuere B, Van der Jeugt F, Willems T, Naessens T, Devreese B, Martens L, Dawyndt P. 2017. High-throughput metaproteomics data analysis with Unipept: A tutorial. J Proteomics.

14. Pundir S, Martin MJ, O'Donovan C. 2016. UniProt Tools. Curr Protoc Bioinformatics 53:1 29 21-15.

15. Kanehisa M, Sato Y, Kawashima M, Furumichi M, Tanabe M. 2016. KEGG as a reference resource for gene and protein annotation. Nucleic Acids Res 44:D457-462.

16. Pham TV, Jimenez CR. 2012. An accurate paired sample test for count data. Bioinformatics 28:i596-i602.

17. Tanca A, Manghina V, Fraumene C, Palomba A, Abbondio M, Deligios M, Silverman M, Uzzau S. 2017. Metaproteogenomics Reveals Taxonomic and Functional Changes between Cecal and Fecal Microbiota in Mouse. Front Microbiol 8:391.

18. Benjamini Y, Hochberg Y. 1995. Controlling the false discovery rate: a practical and powerful approach to multiple testing. J R Statist Soc B:289-300
