# Supplementary figures and images for: Clostridial Butyrate Biosynthesis Enzymes Are Significantly Depleted in the Gut Microbiota of Nonobese Diabetic Mice
Source: mSphere. 2018 Oct 24;3(5):e00492-18. doi: 10.1128/mSphere.00492-18 (PMC6200989; doi:10.1128/mSphere.00492-18)

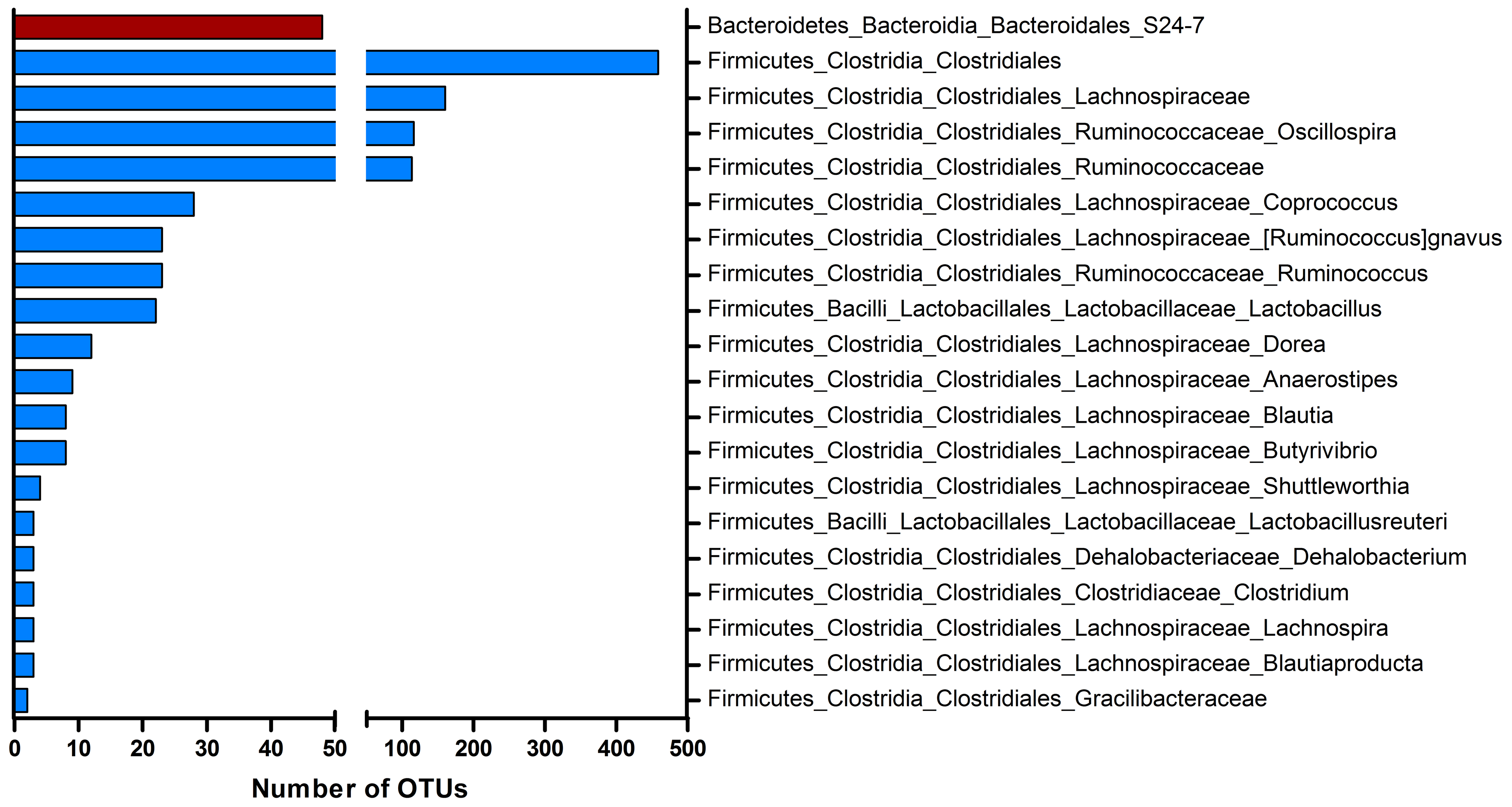

Supplement: FIG S1 [file sph006182677sf1.tif]

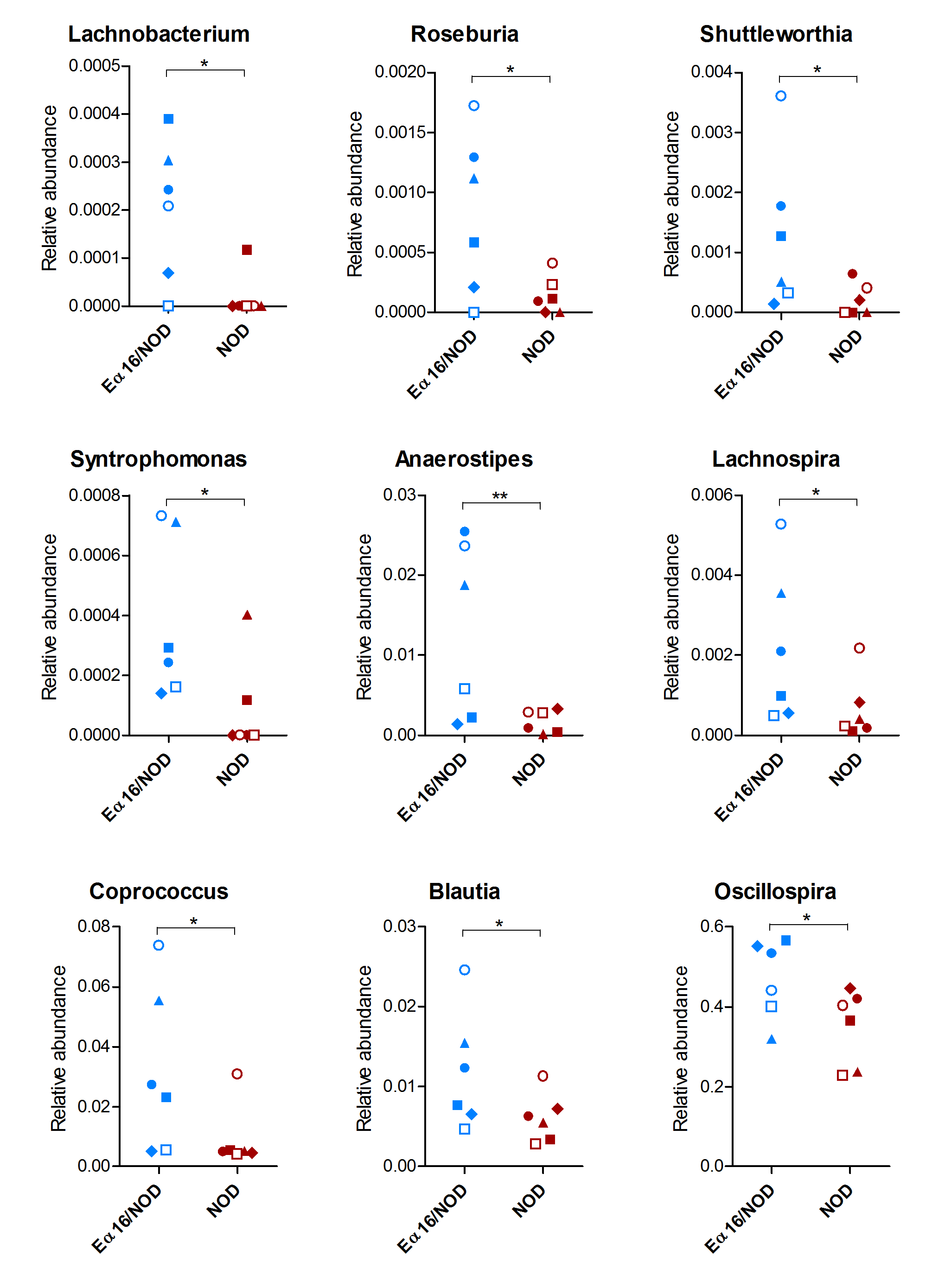

Supplement: FIG S2 [file sph006182677sf2.tif]
